# Successful hemodialysis withdrawal with glucocorticoid treatment after achieving infection control in a case of crescentic IgA-dominant staphylococcus-associated glomerulonephritis

**DOI:** 10.1007/s13730-026-01182-7

**Published:** 2026-09-17

**Authors:** Yuto Nishiyama, Mimiko Matsumura, Yukiko Yasui, Nozomi Omizo, Takahiro Saito, Miho Kawashima, Nayuta Seto, Yuichiro Nagase, Shinichiro Morioka, Daisuke Katagiri, Hideki Takano

**Affiliations:** 1 Department of Nephrology, National Center for Global Health and Medicine, Japan Institute for Health Security, 1-21-1 Toyama, Shinjuku-Ku, Tokyo, 162-8655 Japan; 2 Disease Control and Prevention Center, National Center for Global Health and Medicine, Japan Institute for Health Security, Tokyo, Japan

**Keywords:** Glucocorticoids, Hemodialysis, Glomerulonephritis, *Staphylococcus aureus*

## Abstract

Staphylococcus-associated glomerulonephritis (SAGN) is a subtype of infection-related glomerulonephritis (IRGN) that often affects older adults and can progress rapidly. The role of immunosuppressive therapy in IRGN remains controversial due to the risk of worsening infection and limited evidence from randomized controlled trials. In this report, we present the case of a 70 year-old woman who developed bacteremia, pyogenic sacroiliitis, and multiple iliopsoas abscesses caused by methicillin-susceptible *Staphylococcus aureus*. While receiving pathogen-directed antibiotics, the patient developed acute kidney injury (peak serum creatinine level, 5.48 mg/dL) with gross hematuria and nephrotic-range proteinuria and subsequently required hemodialysis. Renal biopsy revealed endocapillary hypercellularity and cellular-to-fibrocellular crescents with mesangial expansion and hypercellularity. Immunofluorescence tests demonstrated IgA-dominant deposits with C3, and electron microscopy revealed mesangial-to-subendothelial electron-dense deposits, supporting the diagnosis of IgA-dominant SAGN. Glucocorticoid treatment was initiated after confirming regression of the iliopsoas abscesses and objective evidence of infection control, while continuing pathogen-directed antibiotics. Renal function gradually improved, and hemodialysis was discontinued. In patients with crescentic IgA-dominant SAGN with objectively confirmed infection control and progressive renal dysfunction despite adequate antimicrobial therapy, a carefully timed glucocorticoid treatment strategy, with continued antibiotic therapy and close monitoring, may lead to recovery of renal function and hemodialysis withdrawal.

## Introduction

Infection-related glomerulonephritis (IRGN) is an immune-mediated glomerular injury associated with active infection. Although historically considered a post-infectious disease of childhood following streptococcal infection, its epidemiology has shifted toward older adults with comorbidities in whom renal injury often develops while the infection is ongoing rather than after its resolution [1]. Consequently, the term “IRGN” has replaced “post-infectious glomerulonephritis” in contemporary practice. *Staphylococcus aureus* has emerged as the leading pathogen causing IRGN in adults. A distinctive variant, IgA-dominant Staphylococcus-associated glomerulonephritis (SAGN), is characterized by dominant or co-dominant IgA deposition with C3, endocapillary hypercellularity, and frequent crescent formation. SAGN typically affects older adults and may present with rapidly progressive renal dysfunction, nephrotic-range proteinuria, and dialysis dependence [2, 3]. Its pathogenesis is thought to involve infection-driven immune complex formation and complement activation, leading to persistent glomerular inflammation.

Differentiating IgA-dominant IRGN from primary IgA nephropathy is essential because the therapeutic strategies differ substantially. Infection-related cases often have an abrupt onset temporally associated with systemic infection, prominent endocapillary proliferation, and subendothelial deposits on electron microscopy, suggesting an infection-driven process. Current management emphasizes eradication of the underlying infection with appropriate antimicrobial therapy and source control. The Kidney Disease: Improving Global Outcomes (KDIGO) guidelines discourage routine immunosuppressive therapy for IRGN due to the risk of worsening infections and limited supporting evidence [4, 5]. However, a therapeutic dilemma arises in severe cases of crescentic IRGN with progressive renal failure despite documented clinical improvement in infection. In these cases, persistent immune-mediated injuries may contribute to progressive renal dysfunction.

Herein, we report a case of crescentic IgA-dominant SAGN that developed during the treatment of methicillin-susceptible *Staphylococcus aureus* (MSSA) bacteremia. Glucocorticoids were administered only after achieving microbiological and clinical responses, and antimicrobial therapy was continued, leading to renal function recovery and hemodialysis withdrawal. This case highlights the potential role of carefully timed immunosuppression in selected cases of severe IgA-dominant SAGN.

## Case report

**Fig. 1:**
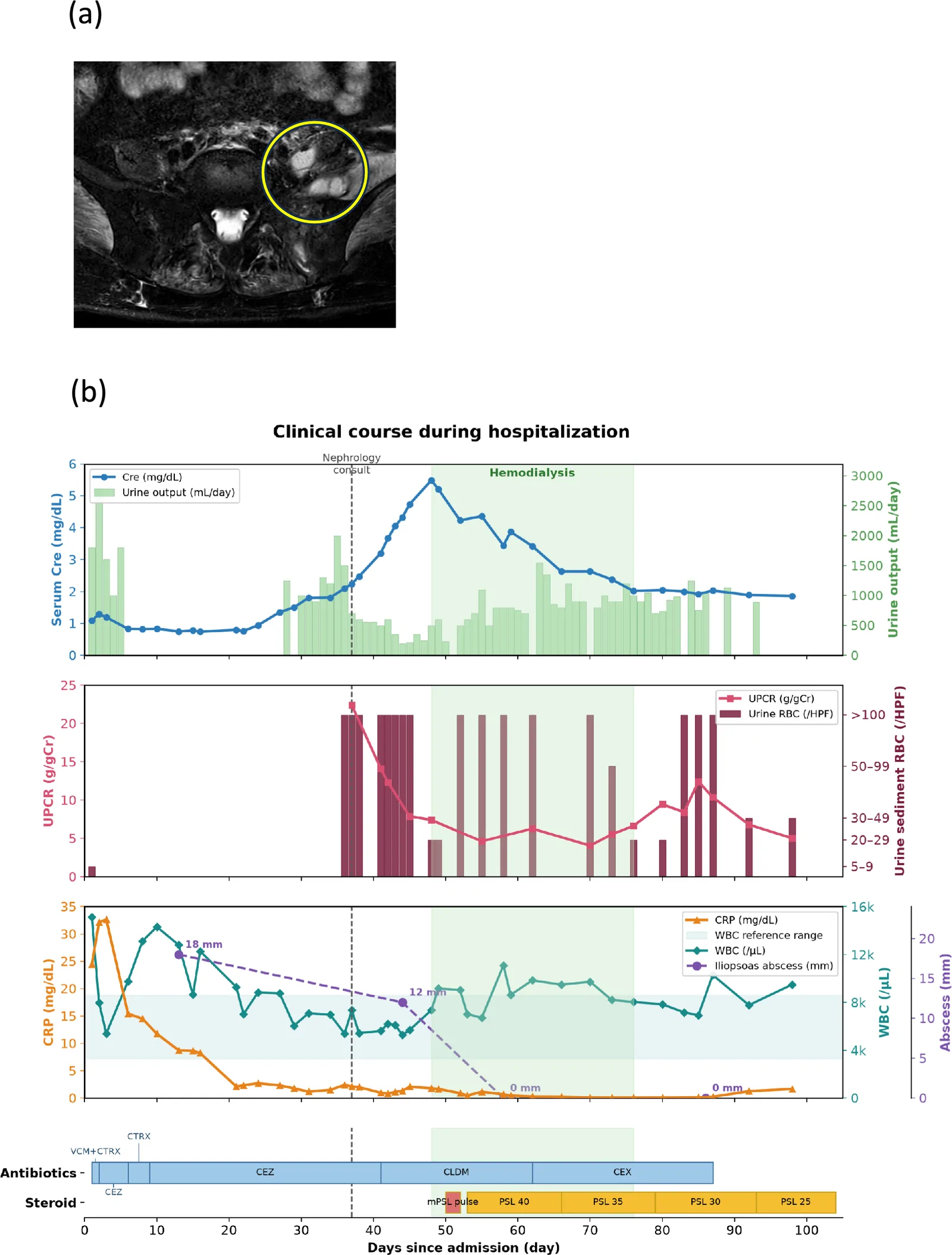
Magnetic resonance imaging findings and clinical course during hospitalization.**a** Axial T2-weighted magnetic resonance image obtained with fat suppression on day 13 shows a hyperintense fluid collection in the left iliopsoas muscle, consistent with an abscess (yellow circle).**b** Clinical course during hospitalization, shown against days since admission. From top: serum creatinine level and daily urine output; urine protein-to-creatinine ratio and semiquantitative urine sediment red blood cell count; serum C-reactive protein level, white blood cell count (shaded band, reference range), and iliopsoas abscess longest dimension on serial magnetic resonance imaging; and antibiotic and glucocorticoid timelines. The green shaded area indicates the hemodialysis period (days 48–76), and the vertical dashed line indicates nephrology consultation (day 37). Urine output and sediment data are plotted only for days with available measurements. CEX, cephalexin; CEZ, cefazolin; CLDM, clindamycin; Cre, serum creatinine level; CTRX, ceftriaxone; mPSL, methylprednisolone; PSL, prednisolone; UPCR, urine protein-to-creatinine ratio; VCM, vancomycin

A 70-year-old woman (body weight at admission, 55.0 kg) with a history of hypertension, schizophrenia, dementia, and breast cancer surgery presented with chills and impaired mobility and was admitted to the infectious disease department. She was independent in activities of daily living, had no smoking or alcohol history, and had no known drug allergies. She had not been prescribed or taken any non-steroidal anti-inflammatory drugs (NSAIDs) before or during hospitalization. Family history was unknown. Blood cultures revealed MSSA. Magnetic resonance imaging (MRI) confirmed pyogenic sacroiliitis and multiple iliopsoas abscesses (the largest measuring 18 × 10 mm in cross-section) (Fig. 1a). Antibiotics were administered sequentially: vancomycin plus ceftriaxone (day 1), cefazolin (days 2–5), ceftriaxone (days 6–8), cefazolin (days 9–40), clindamycin (days 41–61), and cephalexin (days 62–87). Blood cultures became negative by day 3, indicating early microbiological response. Given the Staphylococcus aureus bacteremia, transthoracic echocardiography was performed on day 10 and revealed no valvular vegetations or other findings suggestive of infective endocarditis. No percutaneous abscess drainage was performed because the abscesses were considered too small. Clinical status improved, and inflammatory markers declined (serum C-reactive protein level on day 29, 1.76 mg/dL). Computed tomography (CT) performed on day 16 revealed new bilateral pleural effusions.

Serial MRI evaluations performed on days 13, 44, 58, and 86 demonstrated progressive regression in abscess size; the largest abscess decreased in size from 18 × 10 to 12 × 12 mm in cross-section by day 44 and completely resolved by day 58, with no recurrence on day 86.

**Fig. 2:**
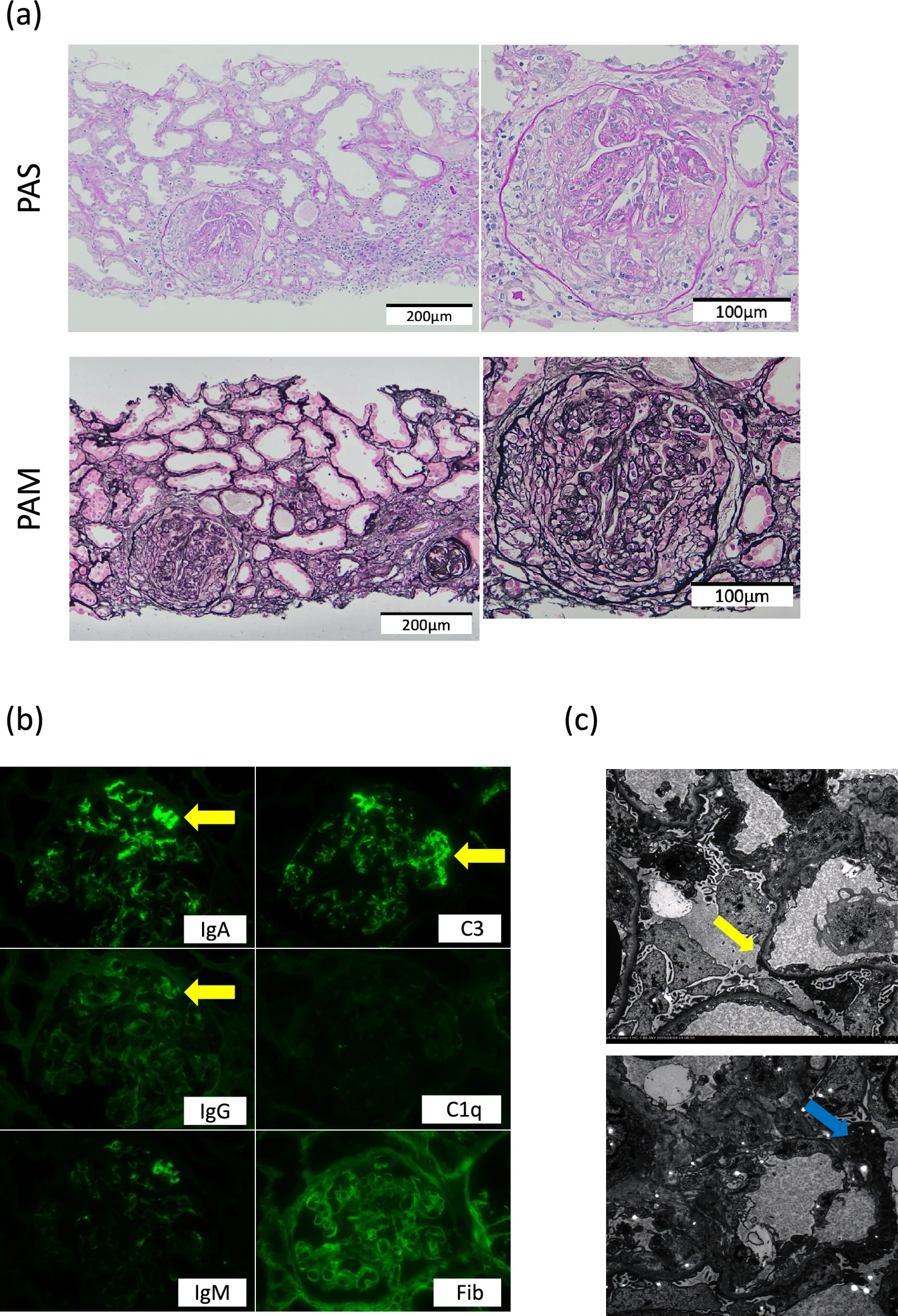
Microscopic findings.**a** Light microscopy images. A glomerulus with a cellular-to-fibrocellular crescent, mesangial expansion, focal double contours of the glomerular basement membrane, and prominent tubulointerstitial inflammatory cell infiltration (PAS stain: original magnification, ×100 and ×200; PAM stain: original magnification, ×100 and ×200). PAM, periodic acid methenamine silver; PAS, periodic acid–Schiff.**b** Immunofluorescence microscopic images (all panels: original magnification, ×400). Yellow arrows indicate representative areas of positive immunofluorescence within the glomeruli.**c** The electron microscopic image. The yellow arrow indicates thinning of the glomerular basement membrane and foot process effacement. (original magnification, ×4000). The blue arrows indicate electron-dense deposits (original magnification, ×5000)

On day 1, serum creatinine level was 1.09 mg/dL, and urinalysis revealed protein 2+, occult blood 3+, and 5–9 red blood cells per high-power field (HPF). Renal function improved (serum creatinine level, 0.78 mg/dL) by day 15, and deteriorated thereafter. Nephrology consultation was conducted on day 37. At the time of consultation, vital signs were as follows: body temperature, 36.8 °C; blood pressure, 152/53 mmHg; pulse rate, 68 beats/min; and respiratory rate, 18 breaths/min. Physical examination revealed bilateral pitting edema of the lower legs; no skin rashes were noted, and no other remarkable findings were observed. The serum creatinine level had increased to 2.24 mg/dL with nephrotic-range proteinuria (urine protein-to-creatinine ratio [UPCR], 22.4 g/gCr) and active urinary sediments (Table 1). Because drug-induced renal injury from cefazolin could not be excluded, cefazolin was switched to clindamycin on day 41. However, renal function continued to decline, and the serum creatinine level increased to 5.48 mg/dL on day 48 when the patient developed oliguria and gross hematuria (Fig. 1b). Hemodialysis (3 times weekly) was initiated on day 48. Serum complement levels did not decrease, and serum autoantibodies were absent. Renal biopsy revealed six glomeruli, including one with global sclerosis and three with cellular-to-fibrocellular crescents. Several glomeruli exhibited mesangial expansion, with mild hypercellularity and occasional double contours of the glomerular basement membrane. The tubulointerstitial compartment showed lymphocytic and plasmacytic infiltration accompanied by moderate interstitial fibrosis and moderate tubular atrophy. The infiltrate was distributed in association with the areas of glomerular injury, without widespread tubulitis, suggesting tubulointerstitial changes secondary to the glomerular disease rather than a primary tubulointerstitial process. Arteriolar hyalinosis was absent, and the vessels showed mild arteriosclerotic changes. Immunofluorescence tests revealed dominant IgA and C3 staining and weakly positive IgM in the mesangium, with trace amounts of IgG, C4d, and fibrinogen; C1q was negative. Electron microscopy revealed mesangial and matrix proliferative changes, with small electron-dense deposits in the paramesangial areas, thinning and segmental dissolution of the glomerular basement membrane, subendothelial deposits, endothelial swelling, and extensive foot process effacement (Fig. 2).

**Table 1:** Laboratory findings on the day of nephrology consultation (day 37)

Parameter	Value	Reference range
*Urinalysis*		
Specific gravity	1.012	1.005 –1.030
Protein (dipstick)	3+	−
Glucose (dipstick)	−	−
Occult blood (dipstick)	3+	−
Urine protein-to-creatinine ratio	22.4 g/gCr	< 0.15 g/gCr
Urine sediment, RBC	> 100/HPF	< 5/HPF
Urine sediment, WBC	10–19/HPF	< 5/HPF
Hyaline casts	3+	Not seen
Granular casts	1+	Not seen
Waxy casts	1+	Not seen
Fatty casts	1+	Not seen
FENa	3.5%	–
FEUN	46.3%	–
Urinary β₂-microglobulin	74,285 μg/L	< 230 μg/L
Urinary NAG	42.9 U/L	< 11.5 U/L
L-FABP/Cr	467.8 μg/gCr	< 8.4 μg/gCr
*Hematology and biochemistry*		
Serum albumin level	2.3 g/dL	3.8–5.2 g/dL
Blood urea nitrogen level	20.9 mg/dL	8–20 mg/dL
Serum creatinine level	2.24 mg/dL	0.46–0.79 mg/dL
eGFR	17.2 mL/min/1.73 m^2^	≥ 60 mL/min/1.73 m^2^
Serum sodium level	141 mEq/L	138–145 mEq/L
Serum potassium level	3.6 mEq/L	3.6–4.8 mEq/L
Serum chloride level	108 mEq/L	101–108 mEq/L
Serum glucose level	81 mg/dL	73–109 mg/dL
HbA1c	5.0%	4.9–6.0%
White blood cell count	7,350/μL	3,300–8,600/μL
Neutrophil count	6,078/μL	1,500–6,500/μL
Monocyte count	338/μL	100–700/μL
Eosinophil count	74/μL	40–400/μL
Basophil count	37/μL	0–100/μL
Hemoglobin level	8.9 g/dL	11.6–14.8 g/dL
Platelet count	263,000/μL	158,000–348,000/μL
INR	1.81	0.9–1.1
APTT	34 s	25–37 s
Fibrinogen	300 mg/dL	200–400 mg/dL
*Serological and immunological tests*		
Serum C-reactive protein level	2.11 mg/dL	< 0.14 mg/dL
Anti-GBM antibody	Negative	< 2.0 U/mL
PR3-ANCA	Negative	< 3.5 U/mL
MPO-ANCA	Negative	< 3.5 U/mL
Serum antinuclear antibody titer	< 40	< 40
Anti-dsDNA antibody	Negative	< 12 IU/mL
Serum IgG level	1,285 mg/dL	870–1,700 mg/dL
Serum IgA level	356.3 mg/dL	110–410 mg/dL
Serum IgM level	61.6 mg/dL	35–220 mg/dL
Serum C3 level	87.0 mg/dL	73–138 mg/dL
Serum C4 level	30.5 mg/dL	11–31 mg/dL
Serum CH50 level	38 U/mL	30–45 U/mL
Serum antistreptolysin O level	37 IU/mL	< 240 IU/mL
Cryoglobulin	Negative	Negative

ANCA, antineutrophil cytoplasmic antibody; APTT, activated partial thromboplastin time; CH50, 50% hemolytic complement activity; dsDNA, double-stranded DNA; eGFR, estimated glomerular filtration rate; FENa, fractional excretion of sodium; FEUN, fractional excretion of urea nitrogen; GBM, glomerular basement membrane; HbA1c, hemoglobin A1c; HPF, high-power field; INR, international normalized ratio; L-FABP, liver-type fatty acid-binding protein; MPO, myeloperoxidase; NAG, N-acetyl-β-D-glucosaminidase; PR3, proteinase 3; RBC, red blood cells; WBC, white blood cells

Given the biopsy-confirmed disease activity, including crescents and endocapillary hypercellularity, and dialysis dependence, glucocorticoid therapy was initiated only after microbiological and clinical responses had been confirmed, while pathogen-directed antibiotic therapy was continued. Intravenous methylprednisolone at 500 mg/day was administered for 3 days on days 50–52, followed by oral prednisolone at 40 mg/day on days 53–65, corresponding to 0.74 mg/kg/day based on the body weight of 53.8 kg at initiation, and then gradually tapered to 25 mg/day by day 98. The MRI (day 44) performed before initiating glucocorticoid treatment (day 50) revealed clear regression of residual abscesses, without radiological indications for drainage. Adequate microbiological and clinical response was considered to have been achieved based on the following objective parameters: negative blood cultures since day 3; the patient had been afebrile since day 10 without recurrence of fever; serum C-reactive protein level had declined from 24.45 mg/dL on admission (peak level, 32.68 mg/dL on day 3) to below 2 mg/dL by day 29 and remained low at 2.11 mg/dL on day 37 (Table 1) and 1.79 mg/dL on day 48; white blood cell count had normalized from 15,120/μL (neutrophils, 95.1%) on admission to 7,350/μL (neutrophils, 75.0%) on day 48; and follow-up MRI demonstrated progressive reduction in iliopsoas abscess size. Glucocorticoids were concurrently administered with clindamycin and subsequently with cephalexin. During hemodialysis, urine output gradually increased from a nadir of 200 mL/d on day 44 to approximately 800–1000 mL/d by day 65, suggesting recovery of native renal function. Hemodialysis was discontinued on day 76, and serum creatinine level remained stable at approximately 1.9–2.0 mg/dL thereafter without dialysis (Fig. 1b). The patient remained clinically stable thereafter and was transferred to a rehabilitation facility on day 104. No adverse events, including infection recrudescence, hyperglycemia, or neuropsychiatric exacerbation, were observed during glucocorticoid therapy. Patient-assessed outcomes were not available due to cognitive impairment.

## Discussion

IRGN in adults is increasingly associated with *Staphylococcus aureus* infection and frequently shows IgA-dominant deposits with crescents, a constellation carrying a high risk of rapid renal decline and dialysis [1, 6]. In the present patient, endocapillary hypercellularity with cellular-to-fibrocellular crescents, IgA-dominant deposits with C3, and mesangial-to-subendothelial electron-dense deposits were all consistent with IgA-dominant SAGN [2, 3]. Because renal involvement in SAGN often coincides with ongoing infection rather than following its resolution [6], the urinary abnormalities and elevated serum creatinine already present on admission (approximately four days after the onset of chills) indicate that glomerular injury began early in the infectious course. The subsequent deterioration requiring dialysis on day 48 is therefore best interpreted as delayed decompensation superimposed on smoldering nephritic activity rather than a late-onset event, an interpretation supported by cellular-to-fibrocellular crescents that reflect a subacute process evolving over several weeks.

The role of glucocorticoids in treating IRGN remains unclear. A randomized controlled trial showed no significant renal benefit from adjunctive glucocorticoids at six months, with more frequent infections in the steroid arm, supporting the prevailing avoidance of routine immunosuppression [5]. However, that trial mainly included “classical” IRGN and not crescentic IgA-dominant SAGN, a phenotype that is underrepresented in prospective data and biologically linked to staphylococcal antigens with superantigen-like properties [6]. Such antigens can drive polyclonal B-cell activation and robust IgA responses, leading to endocapillary hypercellularity and IgA/C3 co-deposition, which may remain histologically active even after systemic infection is controlled. This phenotype closely mimics primary IgA nephropathy, making the distinction essential. In an older adult with recent staphylococcal infection, nephrotic-range proteinuria, endocapillary proliferation with crescents, and strongly positive IgA with C3 but negative C1q—reinforced by the presence of mesangial-to-subendothelial deposits on electron microscopy—favor an infection-related process. These features collectively supported a diagnosis of SAGN rather than primary IgA nephropathy in our patient.

Observational series and case-based studies have reported renal function recovery, including dialysis withdrawal, with glucocorticoid and appropriate antibiotic treatment after documented clinical improvement in infection [7–12]. Although limited by selection and publication biases, they consistently suggest a narrow therapeutic window in which immune-mediated injury persists despite infection control. The present case adds that glucocorticoids were initiated while radiologic collections persisted but were clearly regressing—a circumstance less emphasized in prior reports, which often required complete radiologic resolution or drainage beforehand. Because radiographic resolution of abscesses can lag behind clinical control [13, 14], delaying immunosuppression until complete resolution in a patient with active crescents and dialysis dependence may prolong glomerular inflammation and increase the risk of irreversible scarring. Objective confirmation of infection control was therefore central to our decision: persistently negative blood cultures, declining inflammatory markers, serial imaging showing abscess regression with antibiotics alone, and uninterrupted pathogen-directed therapy together constituted "source control" as a composite judgment [15]. A reduced-dose methylprednisolone pulse (500 mg/day for 3 days) was selected given the residual abscesses, advanced age, and neuropsychiatric comorbidities, balancing anti-inflammatory efficacy against the risk of immunosuppression-related complications.

Generalizability is constrained by the single case report and limited follow-up examinations after transfer to a rehabilitation facility. Because cefazolin was switched to clindamycin shortly before initiating glucocorticoid treatment owing to concerns about possible drug-induced renal injury, the relative contributions of cefazolin withdrawal, glucocorticoids, and the natural course of SAGN to renal function recovery could not be elucidated. However, serum creatinine level continued to increase for a week after cefazolin discontinuation (day 41) until hemodialysis was initiated (day 48), suggesting that cefazolin withdrawal alone could not reverse renal injury. Moreover, biopsy findings are characteristic of immune-mediated injury rather than drug-induced tubulointerstitial nephritis, further supporting a predominant role of SAGN. Criteria for objectively confirming adequate infection control are also not standardized. A pragmatic framework integrating culture kinetics, quantitative inflammatory markers, such as C-reactive protein, and serial cross-sectional imaging seems reasonable but requires prospective validation. Future studies should examine whether brief, biopsy-informed glucocorticoid regimens initiated under predefined criteria for microbiological and clinical response can shorten dialysis or improve renal recovery without increasing infection-related complications.

In conclusion, in an older adult patient with crescentic IgA-dominant SAGN, glucocorticoid therapy was initiated after documented clinical improvement in the infection, leading to hemodialysis withdrawal. For biopsy-confirmed crescentic disease with progressive renal dysfunction despite adequate antimicrobial therapy, a timed glucocorticoid treatment strategy, implemented only after achieving microbiological and clinical responses, while continuing antibiotic therapy, may preserve renal function.

## References

[1] Nasr SH, Radhakrishnan J, D’Agati VD. Bacterial infection-related glomerulonephritis in adults. Kidney Int. 2013;83:792–803. 23302723 10.1038/ki.2012.407

[2] Koo TY, Kim GH, Park MH. Clinicopathologic features of IgA-dominant postinfectious glomerulonephritis. Korean J Pathol. 2012;46:105–14. 23109989 10.4132/KoreanJPathol.2012.46.2.105 PMC3479780

[3] Dhanapriya J, Balasubramaniyan T, Maharajan SP, Dineshkumar T, Sakthirajan R, Gopalakrishnan N, et al. IgA-dominant infection-related glomerulonephritis in India: a single-center experience. Indian J Nephrol. 2017;27:435–9. 29217879 10.4103/ijn.IJN_337_16 PMC5704407

[4] Kidney Disease: Improving Global Outcomes (KDIGO) Glomerular Diseases Work Group. KDIGO 2021 clinical practice guideline for the management of glomerular diseases. Kidney Int. 2021;100:S1–276. 34556256 10.1016/j.kint.2021.05.021

[5] Arivazhagan S, Lamech TM, Myvizhiselvi M, Arumugam V, Alavudeen SS, Dakshinamoorthy S, et al. Efficacy of corticosteroids in infection-related glomerulonephritis-a randomized controlled trial. Kidney Int Rep. 2022;7:2160–5. 36217524 10.1016/j.ekir.2022.07.163 PMC9546739

[6] Takayasu M, Hirayama K, Shimohata H, Kobayashi M, Koyama A. *Staphylococcus aureus* infection-related glomerulonephritis with dominant IgA deposition. Int J Mol Sci. 2022;23:7482. 35806487 10.3390/ijms23137482 PMC9267153

[7] Kapadia AS, Panda M, Fogo AB. Postinfectious glomerulonephritis: is there a role for steroids? Indian J Nephrol. 2011;21:116–9. 21769175 10.4103/0971-4065.82141 PMC3132331

[8] Okuyama S, Wakui H, Maki N, Kuroki J, Nishinari T, Asakura K, et al. Successful treatment of post-MRSA infection glomerulonephritis with steroid therapy. Clin Nephrol. 2008;70:344–7. 18826861 10.5414/cnp70344

[9] Nogueira RF, Oliveira N, Sousa V, Alves R. *Staphylococcus*-induced glomerulonephritis: potential role for corticosteroids. BMJ Case Rep. 2021;14:e237011. 33504520 10.1136/bcr-2020-237011 PMC7843308

[10] Şahin H, Oguz EG, Sökmen FC, Selen T. Is Staphylococcus infection-associated glomerulonephritis overlooked? A case report and literature review. Turk J Nephrol. 2022;31:74–7.

[11] Eswarappa M, Ravi V, Mysorekar V, Gireesh MS. IgA dominant poststaphylococcal glomerulonephritis: complete recovery with steroid therapy. Indian J Nephrol. 2014;24:336–7. 25249734 10.4103/0971-4065.133051 PMC4165069

[12] Anantharaman A, Pandurangan V, Srinivasan D, Joyce D, Balasubramanian S. The pulsing paradox: successful steroid therapy in infection-related glomerulonephritis. Cureus. 2024;16:e64769. 39156284 10.7759/cureus.64769 PMC11329379

[13] Ahn KS, Kang CH, Hong SJ, et al. The correlation between follow-up MRI findings and laboratory results in pyogenic spondylodiscitis. BMC Musculoskelet Disord. 2020;21:428. 32616029 10.1186/s12891-020-03446-4 PMC7333318

[14] Hecquet S, Verhoeven F, Aubry S, Prati C, Wendling D, Chirouze C, et al. Interest of follow-up radiological imaging in patients with pyogenic vertebral osteomyelitis. J Clin Med. 2021;10:2690. 34207268 10.3390/jcm10122690 PMC8233882

[15] Dellinger RP, Levy MM, Rhodes A, Annane D, Gerlach H, Opal SM, Sevransky JE, Sprung CL, Douglas IS, Jaeschke R, Osborn TM, Nunnally ME, Townsend SR, Reinhart K, Kleinpell RM, Angus DC, Deutschman CS, Machado FR, Rubenfeld GD, Webb SA, Beale RJ, Vincent JL, Moreno R; Surviving Sepsis Campaign Guidelines Committee including the Pediatric Subgroup. Surviving sepsis campaign: international guidelines for management of severe sepsis and septic shock: 2012. Crit Care Med. 2013; 41:580–637. 10.1097/CCM.0b013e31827e83af 23353941

